# Lactate/albumin ratio as an independent predictor of mortality in pediatric trauma patients admitted to a pediatric intensive care unit

**DOI:** 10.1007/s00431-026-07208-7

**Published:** 2026-06-29

**Authors:** Ibrahim Bingöl, Kazım Ersin Altınsoy

**Affiliations:** 1 Department of Pediatric Intensive Care, Gaziantep City Hospital, Gaziantep, Turkey; 2 Department of Emergency Medicine, Gaziantep City Hospital, Gaziantep Islam Science and Technology University, Gaziantep, Türkiye

**Keywords:** Pediatric trauma, Lactate/albumin ratio, Neutrophil-to-lymphocyte ratio, Mortality prediction, Pediatric intensive care

## Abstract

Early identification of pediatric trauma patients at high risk of mortality remains challenging despite advances in critical care management. Systemic inflammatory response and subsequent organ dysfunction play a central role in trauma-related mortality. Recently, the lactate/albumin ratio (LAR) and inflammatory biomarkers such as the neutrophil-to-lymphocyte ratio (NLR) have emerged as potential prognostic indicators; however, their independent predictive value in pediatric trauma remains incompletely defined. In this retrospective cohort study, 272 pediatric trauma patients admitted to a tertiary pediatric intensive care unit (PICU) were screened. After excluding 28 patients who died within the first 24 h and 34 patients with incomplete laboratory data, 210 patients were included in the final analysis. Demographic, clinical, and laboratory parameters were collected at admission. The primary outcome was in-hospital mortality. Receiver operating characteristic (ROC) curve analysis and multivariable logistic regression were performed to evaluate independent predictors of mortality. Among 210 patients, 27 (12.9%) died during PICU stay. Non-survivors had significantly higher PRISM III scores and prolonged mechanical ventilation duration. LAR demonstrated the highest discriminatory performance for mortality (AUC 0.918, 95% CI: 0.839–0.978), followed by NLR (AUC 0.900) and PRISM III score (AUC 0.884). In multivariable analysis, LAR emerged as a prominent independent predictor of mortality (OR 12.22, 95% CI: 3.08–48.49, *p* < 0.001), while NLR and PRISM III score remained independently associated with adverse outcomes.

*Conclusion*: The lactate/albumin ratio was independently associated with in-hospital mortality and improved risk discrimination when added to PRISM III in this selected cohort of pediatric trauma patients. As a simple and readily available biomarker, LAR may complement established severity scoring systems and support early risk stratification, although external validation is required before clinical application.

**Table:** 

**What is Known:** • *Serum lactate and inflammatory indices such as the neutrophil-to-lymphocyte ratio are associated with poor outcomes in pediatric trauma, but their independent prognostic value is incompletely defined*. • *Established severity scores like PRISM III predict mortality but may not fully capture the combined effects of tissue hypoperfusion and inflammation*.
**What is New:** • *The lactate/albumin ratio independently predicted in-hospital mortality and showed the highest discrimination (AUC 0.918) among tested markers in PICU-admitted pediatric trauma patients*. • *Adding the lactate/albumin ratio to PRISM III improved risk discrimination, supporting its role as a simple adjunct forearly stratification pending external validation*.

**What is Known:**

• *Serum lactate and inflammatory indices such as the neutrophil-to-lymphocyte ratio are associated with poor outcomes in pediatric trauma, but their independent prognostic value is incompletely defined*.

• *Established severity scores like PRISM III predict mortality but may not fully capture the combined effects of tissue hypoperfusion and inflammation*.

**What is New:**

• *The lactate/albumin ratio independently predicted in-hospital mortality and showed the highest discrimination (AUC 0.918) among tested markers in PICU-admitted pediatric trauma patients*.

• *Adding the lactate/albumin ratio to PRISM III improved risk discrimination, supporting its role as a simple adjunct forearly stratification pending external validation*.

## Introduction

Trauma remains one of the leading causes of admission to pediatric emergency departments and pediatric intensive care units (PICUs) and continues to be a major cause of morbidity and mortality in children worldwide. Despite significant advances in pediatric trauma systems, resuscitation strategies, and intensive care management, trauma-related mortality remains substantial, particularly among patients with severe traumatic brain injury and multisystem trauma [1, 2].

Trauma-related deaths in pediatric patients are commonly described as occurring in early and late phases. Early mortality is primarily related to hypoxia, hypovolemia, and severe traumatic brain injury, whereas late mortality is most often associated with systemic inflammatory response syndrome (SIRS), multiple organ dysfunction syndrome (MODS), sepsis, and acute respiratory distress syndrome (ARDS) [3]. Although improvements in early trauma care have led to better survival in the acute phase, outcomes related to post-traumatic systemic inflammation and organ dysfunction remain suboptimal [4].

Trauma induces a rapid and complex systemic inflammatory response triggered by tissue injury, hypoxia, hypotension, and cytokine release, leading to immune dysregulation and subsequent organ dysfunction. This exaggerated inflammatory response plays a central role in the development of SIRS and MODS, which remain major determinants of late mortality in pediatric trauma patients [5, 6]. Therefore, early identification of pediatric trauma patients at risk for adverse inflammatory responses and organ failure is critical to improving prognosis and guiding intensive care management.

In recent years, increasing attention has focused on readily available laboratory biomarkers that may provide early prognostic information in pediatric trauma patients. Serum lactate has been widely recognized as a marker of tissue hypoperfusion and injury severity and has been shown to be associated with increased mortality in critically ill pediatric trauma populations [7]. More recently, composite biomarkers such as the lactate/albumin ratio (LAR) and inflammatory indices, including the neutrophil-to-lymphocyte ratio (NLR), have emerged as promising predictors of adverse outcomes in pediatric trauma patients admitted to the PICU [7, 8]. However, data regarding their independent prognostic value in pediatric trauma remain limited.

Most previous studies evaluating these biomarkers in pediatric trauma populations were limited by relatively small sample sizes and lacked comprehensive multivariable analyses to identify independent predictors of mortality [6]. Therefore, further studies integrating both clinical parameters and laboratory biomarkers in larger cohorts are needed.

The aim of this study was to identify clinical and laboratory predictors of mortality in pediatric trauma patients admitted to a pediatric intensive care unit, with particular emphasis on evaluating the independent prognostic performance of the lactate/albumin ratio in comparison with established severity scores and inflammatory markers.

## Method

This retrospective cohort study was conducted in the 30-bed Pediatric Intensive Care Unit (PICU) of Gaziantep City Hospital, a tertiary referral center providing advanced pediatric critical care services. Pediatric trauma patients admitted to the PICU between January 2024 and January 2026 were screened for eligibility. During the study period, a total of 272 trauma patients were admitted to the PICU. Of these, 28 patients who died within the first 24 h of admission were excluded in order to minimize bias related to immediate fatal injuries. In addition, 34 patients were excluded due to incomplete laboratory data required for biomarker analysis. Consequently, 210 pediatric trauma patients met the inclusion criteria and were included in the final analysis. Patients aged between 1 month and 18 years who were admitted within 24 h of traumatic injury were eligible. All patients were initially evaluated in the emergency department and subsequently transferred to the PICU when intensive monitoring or organ support was required. Patients with known chronic organ failure, genetic or metabolic disorders, chronic inflammatory diseases, or incomplete medical records were excluded. The study protocol was approved by the Institutional Clinical Research Ethics Committee of Gaziantep City Hospital (Approval No: 441/2026) and was conducted in accordance with the principles of the Declaration of Helsinki.

Clinical and laboratory data were retrieved from the hospital’s electronic medical record system using a standardized data collection form. Recorded variables included demographics (age, sex), trauma characteristics (mechanism, type, and region — isolated versus multisystem, the latter defined as injuries involving two or more organ systems or anatomical regions), and clinical course (requirement and duration of mechanical ventilation, inotropic support, blood product transfusion, and length of PICU stay).

Blood product transfusion was administered according to the institutional pediatric trauma protocol, aligned with the Transfusion and Anemia Expertise Initiative (TAXI) consensus recommendations. Packed red blood cells (PRBC) were transfused for hemoglobin < 7 g/dL in stable patients, < 9 g/dL in hemodynamic instability, ongoing hemorrhage, or traumatic brain injury, and during damage-control resuscitation for severe hemorrhagic shock. Platelets were transfused prophylactically for counts < 20,000/µL, < 50,000/µL for active bleeding or invasive procedures, and < 100,000/µL for central nervous system injury or neurosurgery. Fresh frozen plasma (FFP) was transfused for active bleeding with international normalized ratio (INR) > 1.5, established trauma-associated coagulopathy, or as part of 1:1:1 balanced resuscitation in massive transfusion protocols [9, 10].

Laboratory parameters obtained within the first 24 h of PICU admission were recorded; when multiple measurements were available, the worst value was used. Variables included complete blood count, serum lactate, albumin, LAR, NLR, procalcitonin (PCT), C-reactive protein (CRP), coagulation parameters (PT, APTT, INR), and creatinine. The Pediatric Risk of Mortality III (PRISM III) score was calculated using the worst physiological and laboratory values within the first 24 h, according to established scoring criteria [11].

The primary outcome was all-cause in-hospital mortality during PICU stay. Secondary outcomes were PICU length of stay, requirement and duration of mechanical ventilation, inotropic support, and blood product transfusion.

### Statistical analysis

All statistical analyses were performed using SPSS v29.0 (IBM Corp., Armonk, NY, USA). Normality was assessed by the Shapiro–Wilk test. Continuous variables, expressed as mean ± standard deviation or median (interquartile range) as appropriate, were compared using the Student’s t-test or Mann–Whitney U test. Categorical variables, presented as n (%), were compared using the χ^2^ or Fisher’s exact test. ROC curve analysis was performed for PRISM III and laboratory biomarkers, and the area under the curve (AUC) with 95% confidence intervals was calculated. Optimal cut-off values were determined by the Youden index. Variables with a univariable p-value < 0.10 entered a multivariable logistic regression model; the number of predictors was limited considering the 27 mortality events to prevent overfitting, and adjusted odds ratios (ORs) with 95% CIs were reported. To evaluate whether LAR provided incremental prognostic value beyond PRISM III, the AUCs of PRISM III alone and PRISM III + LAR were compared using the DeLong test [12], and the continuous Net Reclassification Improvement (NRI) and Integrated Discrimination Improvement (IDI) were calculated as described by Pencina and colleagues [13]. Model calibration and overall fit were assessed by the Hosmer–Lemeshow test and Nagelkerke R^2^, respectively, and the standardised mortality ratio (SMR; observed-to-expected deaths) was calculated using the original Pollack equation. Internal validation of the multivariable model was performed by bootstrap resampling (B = 1000 iterations) with optimism correction [14], and apparent and optimism-corrected AUC, Brier score, and calibration slope were reported. A two-sided p-value < 0.05 was considered statistically significant.

## Results

### Study population and baseline characteristics

Of the 210 patients in the final cohort, 27 (12.9%) died during PICU stay. The exclusion of 28 patients who died within 24 h and 34 with incomplete data is detailed in the Methods.

The median age was 119 months (IQR 65–162.8), 143 patients (68.1%) were male, and multisystem trauma was observed in 120 (57.1%), with isolated trauma in 90 (42.9%).

Falls were the most common mechanism (*n* = 73, 34.8%), followed by motor vehicle accidents (*n* = 70, 33.3%), pedestrian injuries (*n* = 38, 18.1%), and assault (*n* = 14, 6.7%); penetrating trauma (*n* = 7, 3.3%), bicycle accidents (*n* = 5, 2.4%), and sports-related injuries (*n* = 3, 1.4%) were less frequent.

Cranial injuries were the most frequently affected region (*n* = 84, 40.0%), followed by thoracic (*n* = 35, 16.7%), abdominal (*n* = 28, 13.3%), and extremity injuries (*n* = 21, 10.0%); multiple anatomical region involvement was present in 42 patients (20.0%).

At admission, the median PRISM III score was 8 (IQR 5–12). Mechanical ventilation was required in 120 patients (57.1%) for a median 1 (0–3) day, inotropic support in 66 (31.4%), and the median PICU length of stay was 6 (4–8) days.

Blood product transfusion was required in a substantial proportion of patients: packed red blood cell (RBC) in 69 (32.9%), platelet in 45 (21.4%), and fresh frozen plasma (FFP) in 44 (21.0%) patients. These findings indicate a notable burden of trauma-associated coagulopathy and hemorrhagic instability. Baseline characteristics are summarised in Table 1.

**Table 1:** Baseline characteristics of pediatric trauma patients (*n* = 210)

Characteristics	Value (*n* = 210)
**Demographics**	
Age (months), median (IQR)	119 (65–162.8)
Male sex, n (%)	143 (68.1)
Female sex, n (%)	67 (31.9)
**Type of Trauma, n (%)**	
Falls	73 (34.8)
Motor vehicle accidents	70 (33.3)
Pedestrian injuries	38 (18.1)
Assault	14 (6.7)
Penetrating trauma	7 (3.3)
Bicycle accidents	5 (2.4)
Sports-related injuries	3 (1.4)
**Region of Trauma, n (%)**	
Cranial injuries	84 (40.0)
Thoracic injuries	35 (16.7)
Abdominal injuries	28 (13.3)
Extremity injuries	21 (10.0)
Multiple region involvement	42 (20.0)
**Clinical Severity and Outcomes**	
PRISM III score, median (IQR)	8 (5–12)
Mechanical ventilation required, n (%)	120 (57.1)
Inotropic support required, n (%)	66 (31.4)
PICU length of stay (days), median (IQR)	6 (4–8)
Overall mortality, n (%)	27 (12.9)

Continuous variables are expressed as median (interquartile range [IQR]); categorical variables are expressed as frequency (percentage). Abbreviations: *IQR* interquartile range, *PICU* pediatric intensive care unit, *PRISM III* Pediatric Risk of Mortality III score

### Comparison between survivors and non-survivors

Non-survivors (n = 27) had significantly higher PRISM III scores than survivors (*n* = 183) at admission (18 [13–23.5] vs. 8 [4–12], *p* < 0.001). Duration of mechanical ventilation (9 [0–14.5] vs. 1 [0–4] days, *p* = 0.002) and PICU length of stay (20 [15–23] vs. 5 [3–7] days, *p* < 0.001) were significantly longer in non-survivors, whereas the requirement for mechanical ventilation, inotropic support, and blood product transfusion did not differ between groups (Table 2).

**Table 2:** Comparison of clinical and laboratory parameters between survivors and non-survivors

Variable	Survivors (*n* = 183)	Non-survivors (*n* = 27)	*p*-value
**Clinical Parameters**			
PRISM III score	8.0 (4.0–12.0)	18.0 (13.0–23.5)	< 0.001
Mechanical ventilation duration (days)	1.0 (0.0–4.0)	9.0 (0.0–14.5)	**0.002**
PICU length of stay (days)	5.0 (3.0–7.0)	20.0 (15.0–23.0)	** < 0.001**
Mechanical ventilation required, n (%)	104 (56.8%)	16 (59.3%)	0.976
Inotropic support required, n (%)	60 (32.8%)	6 (22.2%)	0.378
RBC transfusion required, n (%)	61 (33.3%)	8 (29.6%)	0.870
Platelet transfusion required, n (%)	42 (23.0%)	3 (11.1%)	0.251
FFP transfusion required, n (%)	41 (22.4%)	3 (11.1%)	0.275
**Laboratory Parameters**			
Lactate (mmol/L)	2.1 (1.6–2.6)	4.4 (2.9–6.5)	** < 0.001**
Albumin (g/dL)	3.8 (3.4–4.1)	2.6 (2.3–3.25)	** < 0.001**
Lactate/Albumin Ratio (LAR)	0.56 (0.41–0.71)	1.67 (1.12–2.25)	** < 0.001**
NLR	2.9 (1.85–4.0)	7.2 (4.9–10.4)	** < 0.001**
Procalcitonin (ng/mL)	0.83 (0.46–1.25)	3.31 (1.81–4.96)	** < 0.001**
CRP (mg/L)	6.3 (3.8–8.95)	9.1 (4.5–13.6)	0.054
INR	1.12 (1.0–1.2)	2.06 (1.72–2.44)	** < 0.001**
PT (sec)	13.2 (12.3–14.15)	20.4 (18.3–22.85)	** < 0.001**
APTT (sec)	31.3 (27.95–34.65)	62.5 (48.9–74.7)	** < 0.001**
Creatinine (mg/dL)	0.65 (0.51–0.8)	0.68 (0.45–1.02)	0.412
WBC (10^3^/µL)	10.4 (8.65–12.05)	10.2 (9.2–16.55)	**0.100**
Hemoglobin (g/dL)	11.9 (10.3–13.0)	11.2 (9.9–12.3)	**0.209**
Platelet count (10^3^/µL)	269 (174–326)	259 (164–286)	**0.217**
Sodium (mmol/L)	140.2 (138.0–142.8)	142.2 (136.1–144.65)	0.724
Potassium (mmol/L)	4.4 (4.0–4.7)	4.2 (3.55–4.55)	0.098
Calcium (mg/dL)	9.3 (8.9–9.8)	7.8 (7.6–8.1)	** < 0.001**
Magnesium (mg/dL)	2.07 (1.94–2.22)	1.5 (1.34–1.66)	** < 0.001**
AST (U/L)	48.0 (30.0–64.0)	196.0 (144.0–270.5)	** < 0.001**
ALT (U/L)	36.0 (22.5–52.0)	178.0 (103.5–226.5)	** < 0.001**

Data are presented as median (interquartile range [IQR]). Bold p-values indicate statistical significance (*p* < 0.05). Abbreviations: *ALT* alanine aminotransferase, *APTT* activated partial thromboplastin time, *AST* aspartate aminotransferase, *CRP* C-reactive protein, *INR* international normalized ratio, *LAR* lactate/albumin ratio, *NLR* neutrophil-to-lymphocyte ratio, *PICU* pediatric intensive care unit, *PRISM III* Pediatric Risk of Mortality III score, *PT* prothrombin time, *WBC* white blood cell, *RBC* red blood cells, *FFP* fresh frozen plasma

### Laboratory findings

Admission lactate (4.4 [2.9–6.5] vs. 2.1 [1.6–2.6] mmol/L) and LAR (1.67 [1.12–2.25] vs. 0.56 [0.41–0.71]) were significantly higher, and albumin (2.6 [2.3–3.25] vs. 3.8 [3.4–4.1] g/dL) significantly lower in non-survivors (all *p* < 0.001). Inflammatory markers were elevated in non-survivors: NLR (7.2 vs. 2.9, *p* < 0.001) and procalcitonin (3.31 vs. 0.83 ng/mL, *p* < 0.001), whereas CRP did not reach significance (*p* = 0.054). Coagulation parameters (INR, PT, APTT) and transaminases (AST 196 vs. 48; ALT 178 vs. 36 U/L) were significantly impaired in non-survivors (all *p* < 0.001), while creatinine, white blood cell count, hemoglobin, platelet count, sodium, and potassium did not differ between groups. Calcium and magnesium were significantly lower in non-survivors (both *p* < 0.001). All values are presented in Table 2.

### ROC curve analysis

**Fig. 1:**
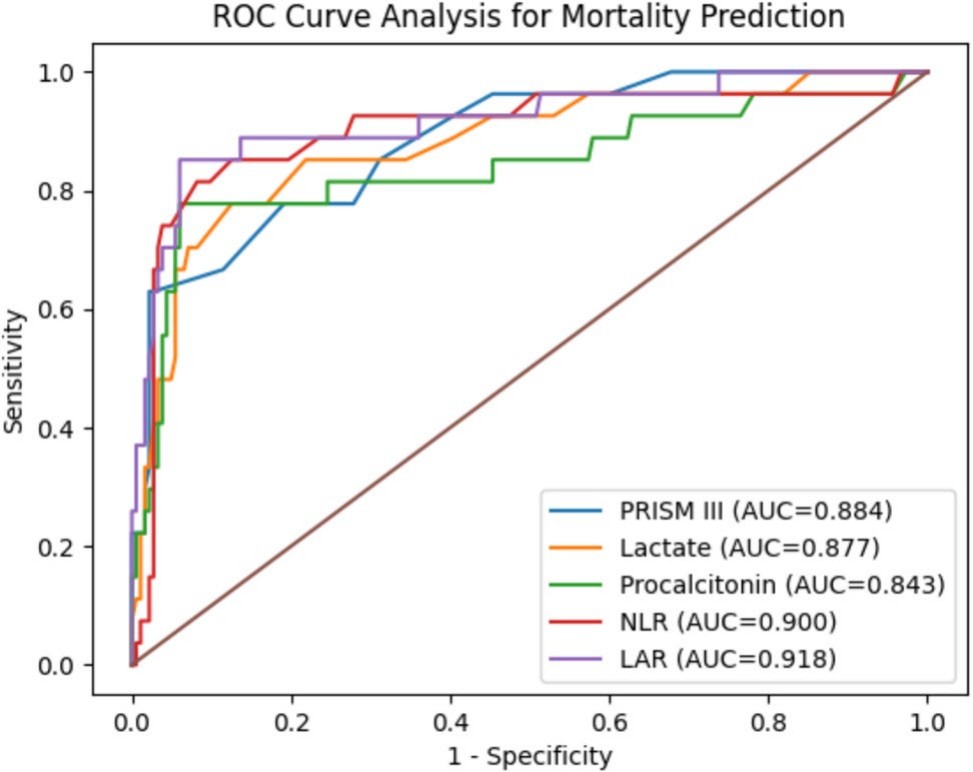
ROC curve analysis for mortality prediction. Abbreviations: AUC, area under the curve; LAR, lactate/albumin ratio; NLR, neutrophil-to-lymphocyte ratio; PRISM III, Pediatric Risk of Mortality III score

ROC curve analysis showed that LAR had the highest discriminatory performance for in-hospital mortality (AUC 0.918, 95% CI 0.839–0.978; cut-off 0.96; sensitivity 85.2%, specificity 94.0%), followed by NLR (AUC 0.900, 95% CI 0.815–0.967; cut-off 4.7), PRISM III (AUC 0.884, 95% CI 0.811–0.946; cut-off 16), lactate (AUC 0.877; cut-off 2.9 mmol/L), and procalcitonin (AUC 0.843; cut-off 1.66 ng/mL). All p-values < 0.001. Inflammatory and metabolic biomarkers — particularly LAR and NLR — demonstrated predictive performance comparable to or exceeding that of the PRISM III score (Table 3, Fig. 1).

**Table 3:** Predictive performance of markers for mortality based on ROC curve analysis

Markers	Cut-off Value	Sensitivity (%)	Specificity (%)	AUC (95% CI)	*p*-value
LAR	0.96	85.2	94.0	0.918 (0.839–0.978)	** < 0.001**
NLR	4.7	81.5	91.8	0.900 (0.815–0.967)	** < 0.001**
PRISM III score	16.0	63.0	97.8	0.884 (0.811–0.946)	** < 0.001**
Lactate	2.9 mmol/L	77.8	87.4	0.877 (0.787–0.947)	** < 0.001**
Procalcitonin	1.66 ng/mL	77.8	94.0	0.843 (0.731–0.934)	** < 0.001**

*AUC* Area under the curve, *LAR* Lactate/Albumin ratio, *NLR* Neutrophil-to-lymphocyte ratio, *PRISM III* Pediatric Risk of Mortality III score

Applying Pollack’s original PRISM III equation to the cohort, the mean PRISM III–predicted mortality was 1.82%, corresponding to 3.82 expected deaths; against 27 observed deaths, the standardized mortality ratio (SMR) was 7.06 (95% CI 4.65–10.27). When the PRISM III–mortality relationship was recalibrated to the study cohort using logistic regression, the model demonstrated good calibration (Hosmer–Lemeshow *p* = 0.68), indicating that the elevated SMR reflects miscalibration of the original equation in pediatric trauma rather than non-linearity of the PRISM–mortality relationship.

### Multivariable logistic regression analysis

Variables with univariable association and good ROC discrimination were entered into a multivariable logistic regression model; LAR was used instead of individual lactate and albumin values to avoid multicollinearity, and variance inflation factor analysis confirmed no concerning multicollinearity (all VIF < 2).

**Fig. 2:**
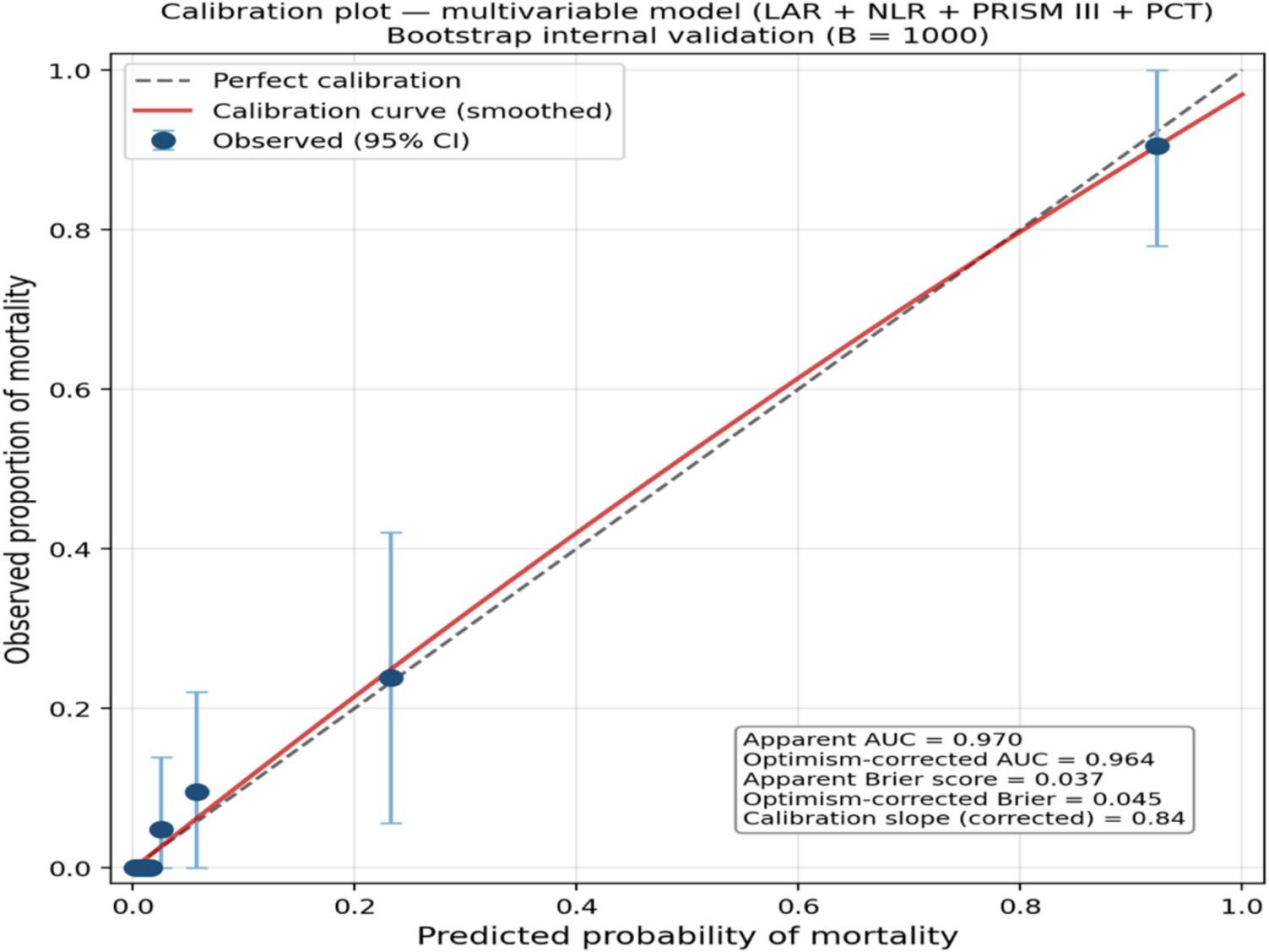
Calibration plot for the multivariable logistic regression model (LAR + NLR + PRISM III + procalcitonin) predicting in-hospital mortality. Abbreviations: CI, confidence interval; LAR, lactate/albumin ratio; NLR, neutrophil-to-lymphocyte ratio; OR, odds ratio; PRISM III, Pediatric Risk of Mortality III score

LAR emerged as a prominent independent predictor of mortality (OR 12.22, 95% CI 3.08–48.49, *p* < 0.001), and NLR (OR 1.30, 95% CI 1.07–1.59, *p* = 0.009), PRISM III (OR 1.19, 95% CI 1.04–1.35, *p* = 0.011), and procalcitonin (OR 1.61, 95% CI 1.01–2.57, *p* = 0.046) also remained independently associated with mortality. The model demonstrated good calibration (Hosmer–Lemeshow χ^2^ = 2.11, *p* = 0.98) and acceptable explanatory power (Nagelkerke R^2^ = 0.74). The AUC of PRISM III combined with LAR (0.946, 95% CI 0.903–0.988) was significantly higher than that of PRISM III alone (0.884, 95% CI 0.811–0.946), with ΔAUC = 0.062 (95% CI 0.017–0.106; DeLong *p* = 0.007). The continuous Net Reclassification Improvement was 1.154 (95% CI 0.806–1.501) and the Integrated Discrimination Improvement was 0.209 (95% CI 0.111–0.307; both *p* < 0.001), confirming significant improvement in both discrimination and reclassification (Table 4). Internal validation by bootstrap resampling (B = 1000) yielded an optimism-corrected AUC of 0.964 (apparent 0.970; mean optimism = 0.007), an optimism-corrected Brier score of 0.045 (apparent 0.037), and a bootstrap-validated calibration slope of 0.84; the calibration plot showed visual agreement between predicted and observed mortality across deciles of predicted risk (Fig. 2).

**Table 4:** Multivariable logistic regression analysis of factors associated with mortality

Variable	Odds Ratio (OR)	95% Confidence Interval (CI)	*p*-value
LAR (Lactate/Albumin Ratio)	12.22	3.08–48.49	** < 0.001**
Procalcitonin	1.61	1.01–2.57	**0.046**
NLR (Neutrophil-to-lymphocyte Ratio)	1.30	1.07–1.59	**0.009**
PRISM III score	1.19	1.04–1.35	**0.011**

Data are derived from a multivariable logistic regression model. Bold *p*-values indicate independent predictors of mortality (*p* < 0.05). Model calibration was assessed using the Hosmer–Lemeshow test, and model fit was evaluated using Nagelkerke R^2^. Internal validation by bootstrap resampling (B = 1000): apparent AUC 0.970, optimism-corrected AUC 0.964, optimism-corrected Brier score 0.045, bootstrap-validated calibration slope 0.84

## Discussion

In our cohort, the median age was 119 months and a male predominance was observed, consistent with previous epidemiological reports of higher trauma exposure among boys [2, 15]. Falls and motor vehicle accidents were the most frequent mechanisms, mirroring patterns reported from developing regions where both domestic falls and road traffic accidents contribute considerably to pediatric trauma burden [16]. This pattern may partially reflect regional demographic characteristics, including high pediatric population density and increasing urbanization. The PRISM III score is widely used to predict mortality in critically ill children; in our cohort, PRISM III was significantly higher among non-survivors and demonstrated good discriminatory ability (AUC 0.884), consistent with prior pediatric critical care studies [11, 17]. However, its moderate sensitivity suggests that biochemical markers may provide complementary prognostic information beyond severity scoring systems.

### Inflammation, organ dysfunction, and clinical course

NLR is a simple marker of systemic inflammation, and elevated NLR has been associated with adverse outcomes in adult trauma populations, particularly in traumatic brain injury [18, 19]; Alimohammadi et al. reported similar associations in pediatric TBI, demonstrating that elevated NLR predicted poor clinical outcomes [20]. Durak et al. reported significantly higher NLR in non-survivors with a cut-off of 3.12 [7], but multivariable analysis could not be performed. In our cohort, NLR was significantly elevated in non-survivors with a higher cut-off (4.7), possibly reflecting variations in injury severity and population characteristics, and remained an independent predictor of mortality in multivariable analysis (OR 1.30, *p* = 0.009).

Pathophysiologically, elevated NLR reflects trauma-induced immune dysregulation characterized by neutrophil-driven innate immune activation and relative lymphocyte suppression, an imbalance associated with systemic inflammatory response and progression to organ dysfunction in critically ill patients [21].

PCT, unlike CRP — which may remain elevated for prolonged periods after trauma — rises earlier and correlates more closely with the magnitude of tissue injury [22, 23], and has been shown to be more sensitive than CRP in adult trauma populations. Durak et al. reported significantly higher PCT in non-survivors, associated with inotropic and mechanical ventilation requirements [7]. In our cohort, PCT was significantly elevated in non-survivors with good discriminatory performance (AUC 0.843) and emerged as an independent predictor of mortality in multivariable analysis (OR 1.61, *p* = 0.046), whereas CRP did not reach statistical significance. These findings support the role of PCT as an early inflammatory biomarker in pediatric trauma, although its predictive value appeared lower than that of LAR.

### Lactate, albumin, and the lactate/albumin ratio

Hyperlactatemia is a well-established marker of tissue hypoperfusion and metabolic stress in trauma patients [24]. In our cohort, lactate levels were significantly higher and albumin significantly lower in non-survivors, reflecting systemic inflammation and reduced physiologic reserve.

LAR integrates both metabolic stress and host reserve into a single parameter. Durak et al. reported significantly higher LAR in non-survivors but could not perform multivariable analysis [7]. In our cohort, LAR demonstrated the highest discriminatory performance among all evaluated markers (AUC 0.918, 95% CI 0.839–0.978; Table 3, Fig. 1) and remained a prominent independent predictor of mortality in multivariable analysis (OR 12.22, *p* < 0.001), even after adjustment for PRISM III, NLR, and procalcitonin (Table 4).

Although LAR has been extensively investigated in sepsis and general ICU populations [25, 26], data in pediatric trauma remain limited. Our findings suggest that LAR integrates both metabolic stress and host reserve, potentially providing a more comprehensive reflection of trauma severity compared with single biomarkers.

A methodological consideration warrants attention: PRISM III is a composite mortality index derived from 17 weighted physiological variables, whereas LAR is a single biochemical parameter; including both as independent predictors may conflate variables of fundamentally different nature. To address whether LAR provides incremental prognostic information beyond PRISM III, we compared PRISM III alone with PRISM III + LAR using the DeLong test and continuous NRI/IDI. The addition of LAR produced significant improvement in discrimination (ΔAUC = 0.062, p = 0.007) and reclassification (NRI 1.154, IDI 0.209; both p < 0.001), indicating that LAR contributes prognostic information beyond that captured by PRISM III and supporting its role as a complementary biomarker rather than a substitute for established severity scoring systems. Reclassification metrics are unstable in small samples; these values are exploratory pending external validation. The standardized mortality ratio observed when applying Pollack’s original equation indicates that PRISM III underestimates mortality in pediatric trauma populations, consistent with prior reports [27]; these findings support incorporating trauma-relevant biomarkers such as LAR alongside established severity scores. This miscalibration coexisted with preserved discrimination (AUC 0.884), so PRISM III still rank-orders risk validly. As early deaths were excluded, the true underestimation is likely greater. Internal validation by bootstrap resampling further demonstrated minimal optimism (ΔAUC = 0.007) and an acceptable calibration slope (0.84), supporting internal robustness of the model. Notably, although coagulation function (INR, PT, APTT) was significantly impaired in non-survivors, baseline hemoglobin and platelet counts did not differ between groups, consistent with trauma-induced coagulopathy driven primarily by coagulation factor consumption and hepatic dysfunction rather than frank anemia or thrombocytopenia; this also explains why the proportional requirement for blood product transfusion did not differ between groups.

Severe trauma initiates a complex inflammatory cascade characterized by endothelial activation, microcirculatory dysfunction, and immune dysregulation, which may progress to MODS [4, 21]. The association observed in our study between elevated inflammatory markers, coagulation abnormalities, organ injury markers, and mortality supports the biological plausibility that exaggerated post-traumatic inflammation contributes to progressive organ dysfunction and adverse outcomes. Although causality cannot be established in this retrospective analysis, the concordance of inflammatory, metabolic, and organ injury markers strengthens the validity of our findings.

### Strengths and limitations

This study has several strengths. First, it provides a comprehensive evaluation of both traditional severity scores and inflammatory biomarkers within the same pediatric trauma cohort. Second, LAR was calculated from admission laboratory values, reflecting early physiological derangement rather than treatment-related changes. Third, multivariable logistic regression identified independent predictors of mortality, and the integration of clinical, biochemical, and coagulation parameters allowed a holistic assessment of trauma-related organ dysfunction.

However, several limitations should be acknowledged. First, the retrospective single-center design may limit generalizability. Second, only admission values were analyzed and dynamic changes in biomarkers were not evaluated; serial measurements might provide additional prognostic insight. Third, the limited number of mortality events (*n* = 27) reduced the statistical power to detect differences in secondary outcomes; the absence of significant associations for the requirement of mechanical ventilation, inotropic support, and blood product transfusion most likely reflects a type II error rather than a true lack of clinical association, and should be interpreted with caution. Fourth, the exclusion of patients who died within the first 24 h allowed a focused evaluation of late-phase inflammatory outcomes and organ dysfunction but inherently restricts generalizability to early-phase mortality and may overlook biomarker performance in the hyperacute stage of pediatric trauma; consequently, the discriminative performance of LAR and other biomarkers should be regarded as specific to patients surviving the initial resuscitation phase. Finally, although the multivariable model demonstrated acceptable performance after internal validation by bootstrap resampling, external validation in independent, larger cohorts remains necessary to confirm clinical utility. With four predictors against 27 events (events-per-variable ≈ 6.8, below the recommended 10), residual overfitting cannot be excluded despite bootstrap validation; the adjusted odds ratios and Nagelkerke R^2^ should be read as cohort-specific. The cut-off values for LAR, NLR, and PRISM III derived in this cohort using the Youden index should be regarded as exploratory and require external validation before being applied as clinical thresholds.

## Conclusion

In this cohort of pediatric trauma patients admitted to the PICU, the lactate/albumin ratio demonstrated good discriminatory performance and emerged as a prominent independent predictor of mortality. Inflammatory markers, particularly the neutrophil-to-lymphocyte ratio and procalcitonin, were also significantly associated with adverse outcomes. These findings suggest that simple and readily available laboratory parameters may complement established severity scoring systems in early prognostic assessment. Further multicenter prospective studies are needed to validate these results and determine their role in clinical decision-making.

## Data Availability

The data supporting the findings of this study can be provided by the corresponding author upon reasonable request.

## Abbreviations

ALT: Alanine aminotransferase
APTT: Activated partial thromboplastin time
ARDS: Acute respiratory distress syndrome
AST: Aspartate aminotransferase
AUC: Area under the curve
CI: Confidence interval
CRP: C-reactive protein
FFP: Fresh frozen plasma
ICU: Intensive care unit
INR: International normalized ratio
IQR: Interquartile range
LAR: Lactate/albumin ratio
MODS: Multiple organ dysfunction syndrome
NLR: Neutrophil-to-lymphocyte ratio
OR: Odds ratio
PCT: Procalcitonin
PICU: Pediatric intensive care unit
PRISM III: Pediatric Risk of Mortality III score
PT: Prothrombin time
RBC: Red blood cells
ROC: Receiver operating characteristic
SIRS: Systemic inflammatory response syndrome
VIF: Variance inflation factor
WBC: White blood cell
